# 

**Published:** 1903-07-04

**Authors:** 


### NOTICE TO CORRESPONDENTS.

All MSS., Letters, Books for Review, and other matters intended for the Editor should be addressed The Editor, "The Hospital," The Hospital Buildings, 28 & 29 Southampton Street, Strand, W.C.

The Editor cannot undertake to return rejected MS., even when accompanied by stamped directed envelope.

**Notice to Advertisers and Subscribers.**

All Advertisements, Orders for Copies of The Hospital, and Business Communications should be addressed to **THE MANAGER, (Not to the Editor),** The Hospital, 28 & 29 Southampton Street, Strand, London, W.C.

The Cost of Subscription, post free, is as follows.—Per week, 3½d.; per quarter, 3s. 9d.; per half-year, 7s. 6d.; per annum, 15s. Foreign and Colonial:—Per annum, 19s. 6d., payable, in all cases, in advance.

**NOTICE.—Vol. XXXIII. of "The Hospital" (October 1902 to March 1903) in handsome cloth binding, will shortly be on sale at the office of this Journal, price 7s. 6d. Cases for binding the Numbers contained in this Volume 1s. 6d. Index to Vol. XXXIII., 2½d., post free.**

**The monthly part for July is now ready. Price 1s., by post 1s. 3d.**

### BOOKS RECEIVED.

A. and C. Black.

"The Confession of Faith of a Man of Science." By Ernst Haeckel.

"Plea for a Simpler Life." By George S. Keith.

Cassell and Co., Ltd.

"Tumours, Innocent and Malignant." New Edition. By J. Bland-Sutton.

### SCRAPS AND GLEANINGS.

AT a recent sale a fine specimen of the orchid odontoglossum crispum Mabel Claes realised £168.

THE number of cremations carried out in Woking last year was 275, as against 273 for the previous year.

DURING 1902, 35,895 British vessels entered British ports from places abroad, against 29,580 foreign vessels.

THE building of the new Workhouse Infirmary at Bideford, which contains 38 beds, has cost upwards of £3,200.

THE recent Saturday collection in aid of the Belfast Royal Victoria Hospital has up to the present produced £642 odd, and further sums are expected to be received.

THE premises of the leading business houses, clubs, and banks in Edinburgh and Glasgow, during the King's visit to Scotland, were decorated by Messrs. Defries, who also did a great deal of the decorating of Hull during the visit of the Prince and Princess of Wales there.

DURING the past year 3,343 patients were treated in the wards of the Dundee Infirmary, exclusive of maternity cases, and of this number 2,584 had been cured and relieved. The total number of patients, including out-patients and maternity cases, treated during the year was 23,320, as against 22,737 during the previous year. The total income for the year was £11,775, which showed an increase of £347 from special donations, and £62 from church collections, the subscriptions and working men's contributions being about the same as during the previous year.

### THE STUDENT OF MEDICINE.

### APPOINTMENTS.

**Henry Russell Andrews**, M.D., B.S.Lond., M.R.C.P.Lond., appointed Assistant Obstetric Physician to the London Hospital. **H. N. C. Atkinson**, M.R.C.S., L.R.C.P.Lond., appointed District Medical Officer of the Melton Mowbray Union. **Robert Beattie**, M.D., M.Ch.R.U.I., appointed Medical Officer of Health for Dewsbury. **A. F. Blake**, M.R.C.S., L.R.C.P.Lond., appointed Medical Officer of the Children's Homes of the Stepney Union. **R. H. Botham**, M.R.C.S.Eng., L.S.A., appointed District Medical Officer of the Teesdale Union. **Harold R. Cross**, L.S.A., appointed Assistant Medical Officer to the West Riding Asylum, Wakefield. **M. H. Gillies**, M.D.Toronto, appointed Clinical Assistant to the Chelsea Hospital for Women. **John Keay**, M.D.Glasg., F.R.C.P. Edin., appointed Medical Superintendent to the Bangour Asylum. **Thomas H. Kellock**, M.D.Camb., B.C., F.R.C.S.Eng., appointed Surgeon to the Hospital for Sick Children, Great Ormond Street. **Wilfred V. M. Koch**, M.D.Edin., appointed Assistant Surgeon to the Medical Department of Hong Kong. **C. T. Lewis**, M.R.C.S., L.R.C.P.Lond., appointed District Medical Officer of the Luton Union. **G. H. Moorhead**, L.R.C.P.&S.I., appointed District Medical Officer of the Bridgwater Union. **Herbert George Parker**, L.R.C.P., F.R.C.S.Edin., appointed Honorary Ophthalmic Surgeon to the Bolton Schools and Workshops for the Blind, and Examiner for the St. John Ambulance Association.

### "The Hospital" Institutional Library.

"Hospitals and Asylums of the World." 4 Vols., with a Portfolio of Plans, complete £12 12s.

"Burdett's Hospitals and Charities, 1902." 5s.

"Burdett's Official Nursing Directory, 1903." 3s. net.

"Cottage Hospitals, General, Fever, and Convalescent." 10s. 6d.

"Uniform System of Accounts for Hospitals and Public Institutions." New Edition. 4s. net.

"Hospital Expenditure: The Commissariat." 2s. 6d.

"A Legal Handbook for the Use of Hospital Authorities." 2s. 6d.

"The Cottage Hospital Case Book or Register of Patients." 10s. and 12s. 6d.

"The Furnishing and Appliances of a Cottage." 6d.

All these are published by the SCIENTIFIC PRESS, Ltd., and may be obtained through any bookseller, or direct from the publishers, 28 and 29 Southampton Street, Strand, London, W.C.

### Administrative Appointment.

ABERDEEN ROYAL INFIRMARY.

The Directors of this Hospital invite applications for the office of DISPENSER.

Salary £90 per annum.

Candidates must have Chemist's Certificate from the Pharmaceutical Society.

Applications, with testimonials, to be lodged with the undersigned not later than noon of WEDNESDAY, 8th July.

JOHN B. FINDLAY,
Clerk and Treasurer.

343 Union Street, Aberdeen,
June 27th, 1903. (95)

### Books for Private Nurses.

### ART OF MASSAGE.

By Mrs. CREIGHTON HALE.

Price **6/-** post free.

Every Nurse should master the "Art of Massage." She will find it useful and remunerative. Mrs. Creighton Hale who has had perhaps more experience as a teacher than any other Masseuse has been able in her book to explain with great clearness all the difficulties the pupil meets with.

### OPHTHALMIC NURSING.

By SYDNEY STEPHENSON.

Price **3/6** net, **3/10** post free.

This is a complete and profusely-illustrated work, which should be in the hands of all Nurses in charge of Eye cases.

### THE CARE OF CONSUMPTIVES.

By W. H. DAW, M.R.C.S.

Price **1/-** post free.

The hygienic treatment of consumptives is now regarded as so important that the trained nurse plays a very necessary part in the care of these invalids. The subject cannot be fully studied in the general hospital, therefore every nurse should add to her knowledge by the use of this admirable little text-book.

### FEVERS AND INFECTIOUS DISEASES.

By Dr. WILLIAM HARDING.

Price **1/-** post free.

This is a valuable introduction to the nursing and practical management of these cases.

### THE POCKET CASE-BOOK.

Price **1/-** post free.

Nurses will find this a concise and convenient little case-book.

### ON PREPARATION FOR OPERATION IN PRIVATE HOUSES.

By STANMORE BISHOP, F.R.C.S.

Price **6d.** post free.

A useful little help and guide to the nurse engaged for an operation case in a private house.

*These books are obtainable of all Booksellers, and are published by*
THE SCIENTIFIC PRESS, LIMITED, 28 & 29 Southampton Street, Strand, London,
*who will send their latest Catalogue of Nursing Text-books post free to any Nurse on application.*

